# Interactive training versus self-driven training in the prediction of colorectal polyp histology by trainees using the NICE classification

**DOI:** 10.1186/s12876-023-02680-z

**Published:** 2023-02-23

**Authors:** Jia Wang, Wei-guang Qiao, Yu-tang Ren, Yu Chen, Wei Gong

**Affiliations:** 1 Department of Gastroenterology, The First Affiliated Hospital, Xi’an Medical University, Xi’an, 710077 Shaanxi China; 2 Department of Gastroenterology, Nanfang Hospital, Southern Medical University, Guangzhou, 510515 Guangdong China; 3 Department of Gastroenterology, Beijing Tsinghua Changgung Hospital, School of Clinical Medicine, Tsinghua University, Beijing, China; 4 Department of Gastroenterology, Nanhai Hospital, Southern Medical University, Foshan, Guangdong China; 5 Department of Gastroenterology, Shenzhen Hospital of Southern Medical University, Shenzhen, Guangdong China

**Keywords:** Interactive training, Video-based self-learning, NICE classification, Endoscopists

## Abstract

**Background:**

The COVID-19 pandemic has impacted endoscopic training of the Narrow Band Imaging International Colorectal Endoscopic (NICE) classification, which could accurately predict pathology of colorectal polyps. This study aimed to evaluate the diagnostic performance by trainees of self-driven training vs. interactive training in the prediction of colorectal polyp histology.

**Methods:**

This was a prospective randomized controlled study at five academic centers from January 1, 2021 to May 31, 2021. Trainees with no previous formal training of narrow band imaging or blue light imaging for prediction of colorectal polyp histology were randomly allocated to the self-driven training group or interactive training group. Before and after the training, all trainees were given 20 selected cases of colorectal polyp for testing. Their diagnostic performance was analyzed.

**Results:**

Overall, the two training groups showed similar accuracy of NICE classification (79.3% vs. 78.1%; P = 0.637), vessel analysis (77.8% vs. 77.6%, P = 0.939), and surface pattern analysis (78.1% vs. 76.9%, P = 0.616). The accuracy of color analysis in the interactive training group was better (74.4% vs. 80.0%, P = 0.027). For high-confidence predictions, the self-driven training group showed higher accuracy of NICE classification (84.8% vs. 78.7%, P < 0.001) but no difference for analysis of color (79.6% vs. 81.0%), vessel pattern (83.0% vs. 78.5%), and surface pattern (81.8% vs. 78.5%).

**Conclusions:**

Overall, self-driven training showed comparable accuracy of NICE classification, vessel pattern, and surface pattern to interactive training, but lower accuracy of color analysis. This method showed comparable effectiveness and is more applicable than interactive training. It is worth spreading during the COVID-19 pandemic.

*Trial registration* Name of the registry: Chinese Clinical Trial Registry, Trial registration number: ChiCTR2000031659, Date of registration: 06/04/2020, URL of trial registry record: http://www.chictr.org.cn/showproj.aspx?proj=51994

## Introduction

The Narrow Band Imaging International Colorectal Endoscopic (NICE) classification method was proposed in 2012 and expanded in 2013; it is a simple endoscopic classification to accurately predict pathology of colorectal polyps [1, 2]. For NICE classification, types 1, 2, and 3 are correlated with the histopathological findings of hyperplastic polyp and sessile serrated polyp, adenoma/superficial submucosal invasive cancer, and deep submucosal invasive cancer, respectively [3]. Therefore, differentiation of colorectal polyps during endoscopy using the NICE classification could predict histopathology [3]. Training physicians in NICE classification to differentiate and diagnose the nature of colorectal polyps is of great importance.

However, the COVID-19 pandemic has impacted numerous facets of endoscopists’ lives, including gastrointestinal endoscopy, inpatient consults, outpatient clinics, educational conferences [4], and endoscopic training [5]. To limit the spread of COVID-19 and decrease the impact on trainee confidence due to the reduction in endoscopy case volume, the suspension of interactive training was considered to be justified [6, 7].

Compared to interactive training, video-based or web-based training is easily accessible and efficient [8], and eliminates contact with other trainees, which decreases the risk of infection [9]. According to published reports, video-based or web-based training shows satisfactory diagnostic ability of trainees in predicting colorectal polyps compared to interactive training. In the study by Khan et al. [10] there was no difference in the overall accuracy of histology characterization (83.4% vs. 87.2%; P = 0.19), sensitivity (85.6% vs. 88.5%; P = 0.4), and specificity (79.5% vs. 84.9%; P = 0.31) between interactive training and computer-based self-learning. In Smith et al. [11] the sensitivity of the web-based self-training group compared with the didactic group was 72% versus 83% (P < 0.0005), and the accuracy was 66.1% versus 69.1% (P = 0.275).

This study aimed to evaluate (i) the diagnostic performance of self-driven training vs. interactive training in the prediction of colorectal polyp histology by trainees using the NICE classification and (ii) the subgroup analysis of the NICE classification between self-driven training and interactive training.

## Methods

### Study design

**Fig. 1:**
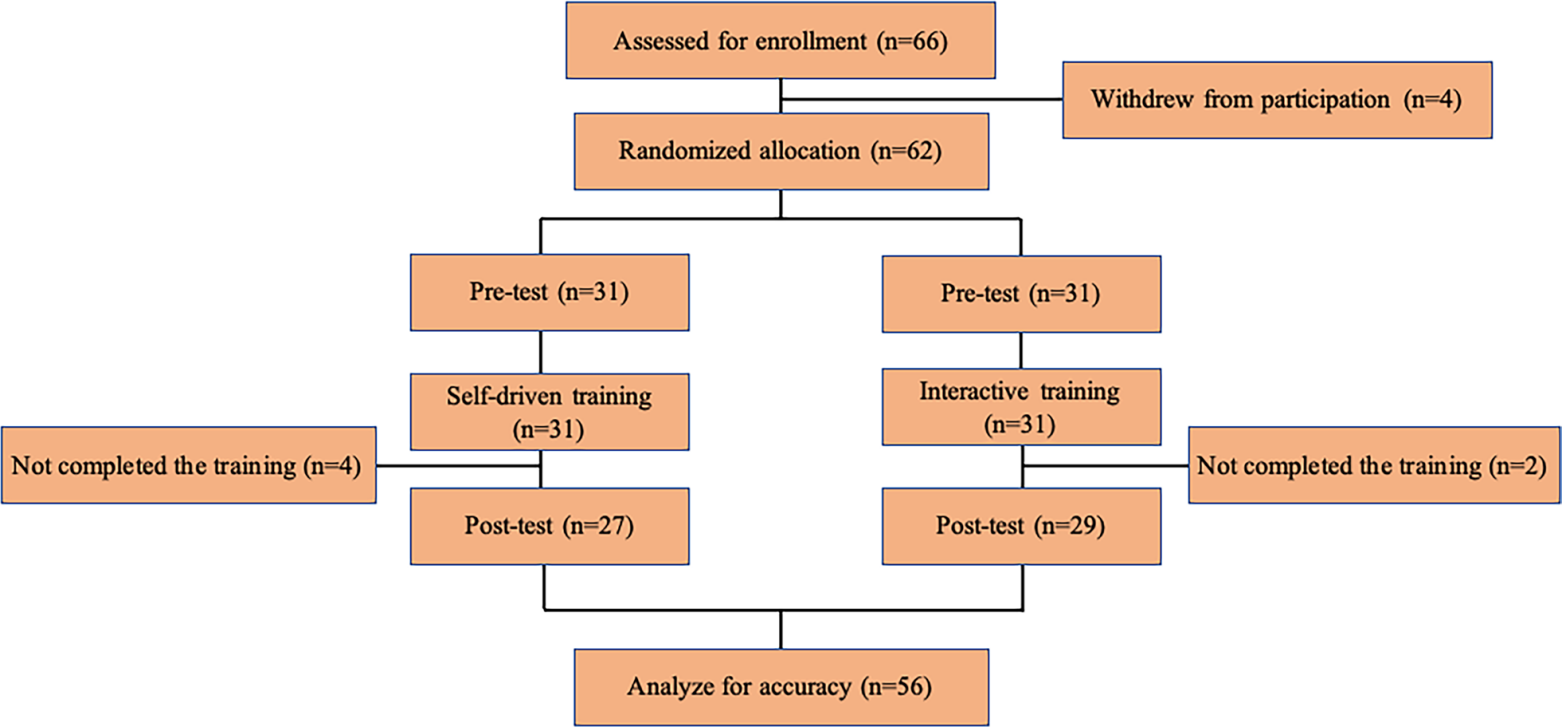
Flow chart of enrolled patient lesions. NBI: narrow band imaging; BLI: blue light imaging; NICE classification: the NBI International Colorectal Endoscopic classification

This research was conducted as a prospective randomized controlled study at five university and academic centers in China from January 1, 2021 to May 31, 2021. Trainees with no previous formal training in the use of Narrow band imaging (NBI) or blue light imaging (BLI) for prediction of colorectal polyp histology using the NICE classification were invited to participate. We enrolled trainees (i) with less than 3 years of colonoscopic experience; (ii) with no previous formal training in the use of NBI or BLI for prediction of colorectal polyp histology; and (iii) with no previous formal training of the NICE classification. Participating trainees provided written informed consent, as they were regarded as the study subjects. They were randomly allocated to either the self-driven training group or the interactive training group using a computerized random number (Fig. 1). The study was preregistered at the Chinese Clinical Trial Registry (Trial registration number: ChiCTR2000031659, Date of registration: 06/04/2020). All methods in this study were carried out in accordance with the Declaration of Helsinki and had been approved by the China Ethics Committee of Registering Clinical Trials approved this study (ChiECRCT20200077).

### Interactive training

The classroom interactive training session was conducted as a group for all trainees randomized to this group. The training slides for the NICE classification used in the study were made by Wei-guang Qiao, and the training video was recorded by Yu-tang Ren. Three endoscopists with experience in NBI or BLI for the NICE classification (Si-lin Huang, and Fa-chao Zhi) reviewed the slides and video.

**Fig. 2:**
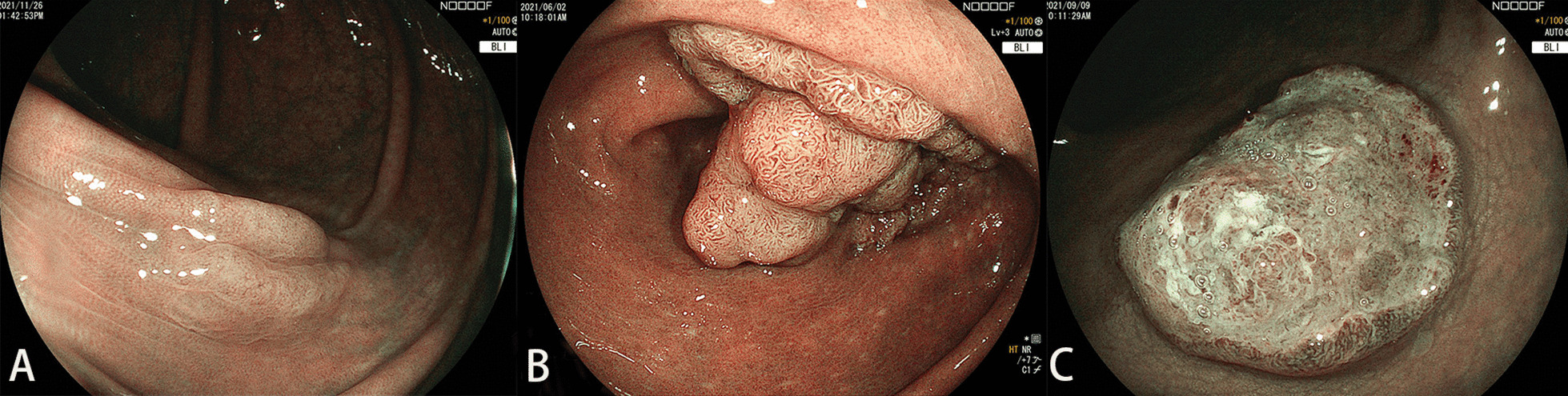
NICE classification. Type 1 featured same or lighter color than background, none or isolated lacy vessels coursing across the lesion with dark or white spots of uniform size, or homogeneous absence of pattern (**A**). Type 2 featured browner color relative to the background (verify color arises from vessels), brown vessels surrounding white structures, oval/tubular or branched white structures surrounded by brown vessels (**B**). Types 3 featured brown to dark brown relative to the background (sometimes patchy whiter areas), area(s) of disrupted or missing vessels, amorphous or absent surface pattern (**C**)

For this training, the trainees could review the training slides and the video once. The training slides contained the key factors for diagnosis via the NICE classification, including the color, the vessel pattern, and the surface pattern (Fig. 2). Type 1 featured a same or lighter color than the background, none or isolated lacy vessels coursing across the lesion with dark or white spots of uniform size, or homogeneous absence of pattern. Type 2 featured browner color relative to the background, brown vessels surrounding white structures, or oval/tubular or branched white structures surrounded by brown vessels. Type 3 featured brown to dark brown relative to the background (sometimes patchy whiter areas), area(s) of disrupted or missing vessels, or amorphous or absent surface pattern. During the training, the trainees were allowed to ask questions. Areas of confusion that could lead to erroneous interpretation were discussed. The training session took a total of 40 min.

### Self-driven training

The trainees in the self-driven training group were provided with a training video with a voice-over recorded by Yu-tang Ren and training slides provided by Wei-guang Qiao, which were similar to those that were used for teaching in the interactive training group. They reviewed the teaching material individually. The trainees could view the slide presentation and training videos at any time within a 40 min timeframe. To make sure the training materials were actually accessed and reviewed by the trainees, a slide with a QR code used for after-training tests was set as the last page.

### Before-training test and after-training test

Before and after the interactive training or self-driven training, all trainees were given a QR code with 20 selected cases of colorectal polyp under BLI for testing. To decrease investigator bias, the investigators who were in charge of the research design were forbidden to be the test scorers. For control measures, the test scorers were blinded to the randomization. The trainees reviewed these images and made a diagnosis by entering the judgement of the NICE classification, the color, the vessel pattern, the surface pattern, and confidence in diagnosis (low or high). All the images were retrieved during standard care without any interventions or exposure of the patient’ personal information. These images of polyps were obtained using high-definition colonoscopes (EC-L590ZW, EC-L590ZP, EC-760ZP-V/M; Fujifilm Co). The polyps in the collected images in the before-training tests and the after-training tests had been resected and evaluated by pathology for histology confirmation.

### Sample size

We used HyLown Consulting (2019 HyLown Consulting LLC, Atlanta, GA) to calculated the sample size. Under the assumption that the accuracy in the self-driven training group would be 70% compared with 78% in the didactic group, a sample size of 481 observations was needed to detect this 10% difference in accuracy between the two groups with 80% power and alpha of 0.05, given that we had 20 cases for tests and a drop rate of 0.05. A sample size of 26 trainees in each group was needed.

### Data analysis

Continuous variables were summarized by the mean, and standard deviation was compared using Student’s *t*-test. Categorical variables were expressed as a proportion or percentage and compared using the Pearson chi-square test. The accuracy of the NICE classification, the color, the vessel pattern, and the surface pattern for each group of different training phases were calculated and compared. Statistical significance was determined by P values < 0.05. SPSS statistical software (version 20.0, SPSS Inc., Chicago, IL, USA) was used.

## Results

### Characteristics of the trainees

Thirty-one endoscopists were allocated to the interactive training group and self-driven training group in the before-training phase, respectively. There were no statistical differences in sex, age, education, colonoscopic experience, magnifying endoscopic experience, or confidence level. The data of 29 endoscopists in the interactive training group and 27 endoscopists in the self-driven training group were collected after training, respectively. There were four trainees in the self-driven training group, and two trainees in the interactive training group did not complete the training (Fig. 1). The baseline characteristics of all the participants are shown in Table 1.

**Table 1:** Baseline characteristics of participants

	Interactive training 31 (50)	Self-driven training 31 (50)	*P*
Sex, n (%)			0.799^a^
Male	16 (48.5)	17 (51.5)	
Female	15 (51.7)	14 (48.3)	
Age, y (Mean Std. Deviation)	35.61 + 4.51	36.23 + 5.94	0.649^b^
Education, n (%)			0.798^a^
Master or Doctor	18 (51.4)	17 (48.6)	
Bachelor	13 (48.1)	14 (51.9)	
Colonoscopic experience, n (%)			0.075^a^
< 500	18 (62.1)	11 (37.9)	
** ≥ **500	13 (39.4)	20 (60.6)	
Magnifying endoscopic experience, n (%)			0.075^a^
< 100	18 (62.1)	11 (37.9)	
** ≥ **100	13 (39.4)	20 (60.6)	
Confidence level, n (%)			1.000^a^
High	12 (50.0)	12 (50.0)	
Low	19 (50.0)	19 (50.0)	

^a^Pearson’s chi-square test

^b^Student’s *t*-test

### Diagnostic performance between the interactive training group and the self-driven training group

Overall, the two training groups showed similar accuracy of NICE classification (71.9% vs. 71.6%, P = 0.900 for the before-training phase; 78.1% vs. 79.3%; P = 0.637 for the after-training phase), vessel analysis (69.8% vs. 66.8%, P = 0.246 for the before-training phase; 77.6% vs. 77.8%, P = 0.939 for the after-training phase), and surface pattern analysis (69.8% vs. 67.7%, P = 0.426 for the before-training phase; 76.9% vs. 78.1%, P = 0.616 for the after-training phase). For the after-training phase, the accuracy of color analysis in the interactive training group was better (80.0.1% vs. 74.4%, P = 0.027).

For high-confidence predictions, there was no difference in the accuracy of histology characterization between the two training groups for the before-training phase (77.4% vs. 73.6%, P = 0.270). For the after-training phase, the two training groups showed similar color analysis (81.0% vs. 79.6%, P = 0.601), vessel analysis (78.5% vs. 83.0%, P = 0.091), and surface pattern analysis (78.5% vs. 81.8%, P = 0. 220) but higher accuracy of NICE classification (73.6% vs. 84.4%, P < 0.001) in the interactive training group.

For low-confidence predictions, the interactive training group was better in the accuracy of NICE classification (75.6% vs. 61.8%, P = 0.027), color analysis (75.5% vs. 56.9%, P = 0.003), and vessel analysis (73.6% vs. 60.2%, P = 0.032). The performance characteristics for predicting colorectal polyp histology in both groups are shown in Table 2.

**Table 2:** Comparisons of NICE ween interactive training and self-driven training

	Before training			After training		
	Interactive training	Self-driven training	*P*	Interactive training	Self-driven training	*P*
Total	n = 620	n = 620		n = 580	n = 540	
Accuracy, n (%)	446 (71.9)	444 (71.6)	0.900^a^	453 (78.1)	428 (79.3)	0.637^a^
Color, n (%)	416 (67.1)	403 (65.0)	0.436^a^	464 (80.0)	402 (74.4)	0.027^a^
Vessel, n (%)	433 (69.8)	414 (66.8)	0.246^a^	450 (77.6)	420 (77.8)	0.939^a^
Surface pattern, n (%)	433 (69.8)	420 (67.7)	0.426^a^	446 (76.9)	422 (78.1)	0.616^a^
High confidence level	345 (55.6)	292 (47.1)	0.003^a^	474 (81.7)	417 (77.2)	0.062^a^
Accuracy, n (%)	267 (77.4)	215 (73.6)	0.270^a^	373 (78.7)	352 (84.8)	0.029^a^
Color, n (%)	262 (75.9)	207 (70.9)	0.149^a^	384 (81.0)	332 (79.6)	0.601^a^
Vessel, n (%)	272 (78.8)	212 (72.6)	0.066^a^	372 (78.5)	346 (83.0)	0.091^a^
Surface pattern, n (%)	263 (76.2)	218 (74.7)	0.645^a^	372 (78.5)	341 (81.8)	0.220^a^
Low confidence level	275 (44.4)	328 (52.9)	0.003^a^	106 (18.3)	123 (22.8)	0.062^a^
Accuracy, n (%)	179 (65.1)	229 (69.8)	0.217^a^	80 (75.5)	76 (61.8)	0.027^a^
Color, n (%)	154 (56.0)	196 (59.8)	0.352^a^	80 (75.5)	70 (56.9)	0.003^a^
Vessel, n (%)	161 (58.5)	202 (61.6)	0.448^a^	78 (73.6)	74 (60.2)	0.032^a^
Surface pattern, n (%)	170 (61.8)	202 (61.6)	0.953^a^	74 (69.8)	81 (65.9)	0.523^a^

^a^Pearson’s chi-square test

### Subgroup analysis of diagnostic performance between the interactive training group and the self-driven training group

There was no statistical difference for the accuracy of NICE classification between the two groups for all training phases (Table 3). For the after-training phase, the male trainees (79.7% vs. 71.0%, P = 0.014), the trainees with experience less than 500 cases (80.45% vs. 70.0%, P = 0.008), and the trainees with magnifying endoscopic experience less than 100 cases (80.71% vs. 72.08%, P = 0.020) in the interactive training group had better accuracy of color analysis (Table 3). For the before-training phase, the trainees with experience less than 500 cases (71.1% vs. 63.2%, P = 0.047) in the interactive training group showed better accuracy of vessel pattern analysis (Table 3). No other statistical difference was found for the accuracy of vessel pattern between the two groups (Table 3). No statistical difference for the accuracy of surface pattern was found between the two groups regardless of the training phases (Table 3).

**Table 3:** Comparisons of subgroup analysis between interactive training and self-driven training (P value)

	NICE		Color		Vessel pattern		Surface pattern	
	Before training	After training	Before training	After training	Before training	After training	Before training	After training
*Sex*								
Male	0.939	0.923	0.952	0.014	0.896	0.337	0.468	0.848
Female	0.990	0.354	0.225	0.650	0.070	0.205	0.709	0.551
*Education*								
Master or Doctor	0.138	0.706	0.362	0.138	0.057	0.979	0.515	0.774
Bachelor	0.133	0.825	0.979	0.090	0.630	0.915	0.701	0.674
*Colonoscopic experience*								
< 500	0.711	0.761	0.289	0.008	0.047	0.497	0.061	0.663
** ≥ **500	0.774	0.307	0.524	0.881	0.856	0.428	0.601	0.235
*Magnifying endoscopic experience*								
< 100	0.641	0.869	0.485	0.020	0.172	0.857	0.106	0.833
** ≥ **100	0.380	0.425	0.065	0.376	0.255	0.770	0.659	0.379

^a^Pearson’s chi-square test

## Discussion

Prediction of the pathology of colorectal polyp before management is of great importance. In China, magnified colonoscopy is not widely available. The NICE classification is a reliable indicator of pathology for colorectal lesions independent of magnified colonoscopy [12]. Therefore, it could be applied widely. According to the NICE classification, type 1 lesions, which are suspected as hyperplastic polyp and sessile serrated polyp, could be followed up or endoscopic resected. Type 2 lesions, suspected as adenoma/superficial submucosal invasive cancer, should be removed by endoscopic treatment, and Type 3 lesions, suspected of deep submucosal invasive cancer, should be removed by surgical treatment [3]. To popularize the application of the NICE classification, training is crucial.

According to our research, interactive training and self-driven training were both found to be reliable training methods. Compared with the before-training phase, both groups showed higher accuracy of NICE classification (71.9% vs. 78.1% for the interactive training group; 71.6% vs. 79.3% for the self-driven training group), higher accuracy of color analysis (67.1% vs. 80.0% for the interactive training group; 65.0% vs. 74.4% for the self-driven training group), higher accuracy of vessel analysis (69.8% vs. 77.6% for the interactive training group; 66.8% vs. 77.8% for the self-driven training group), and higher accuracy of surface pattern (69.8% vs. 76.9% for the interactive training group; 67.7% vs. 78.1% for the self-driven training group). Based on our research, both interactive training and self-driven training could improve the diagnostic accuracy of trainees.

The main advantages of interactive training is the opportunity for discussion with trainees and experts. However, interactive training is currently limited due to the COVID-19 pandemic. Healthcare providers working at an endoscopy center should receive appropriate education and training on infection control measures, including hand hygiene and use of personal protective equipment [13, 14], which has led to limitations in endoscopic volumes and endoscopic training [15, 16] and high rates of anxiety and burnout [4]. Compared to interactive training, whether self-driven training could offer a reliable education method to improve trainees’ skills as a salvage measure remains unclear.

Based on our research, there were no statistical difference in the overall accuracy of NICE classification (78.1% vs. 79.3%), the accuracy of vessel analysis (77.6% vs. 77.8%), and the accuracy of surface pattern analysis (76.9% vs. 78.1%,) in the after-training phase, except for higher accuracy of color analysis in the interactive training group (80.0.1% vs. 74.4%, P = 0.027). For high-confidence predictions in the after-training phase, the interactive training group showed higher accuracy of NICE classification (73.6% vs. 84.4%, P < 0.001) but no difference for analysis of color (81.0% vs. 79.6%), vessel pattern (78.5% vs. 83.0%), and surface pattern (78.5% vs. 81.8%). The self-driven training group showed lower accuracy of color analysis for overall analysis and lower accuracy of NICE classification for high-confidence predictions compared to the interactive training group. Nonetheless, the diagnostic performance of self-driven training in the prediction of colorectal polyp histology by trainees using the NICE classification is practicable and acceptable.

Similar results were found in the research by Khan et al. [10] There was no difference in overall accuracy of histology characterization between interactive training and computer-based self-learning (83.4% vs. 87.2%; P = 0.19). For high-confidence predictions, the accuracy (85.7% vs. 93.9%) was higher in the self-learning group. According to the research by Smith et al. [11] when using NICE, the sensitivity of the didactic group was better (72% vs. 83%, P < 0.0005), but accuracy was comparable (66.1% vs. 69.1%, P = 0.275). Therefore, self-driven training is an effective method to improve the diagnosis performance of trainees using the NICE classification, and it can be used during the COVID-19 pandemic.

Sensitivity analysis and specificity analysis were analyzed in the previous researches by Khan et al. [10], Smith et al. [11], and Allen et al. [17]. Our research may more attention on color analysis, vessel analysis and surface pattern analysis. According to this research, the two training groups showed similar accuracy of NICE classification (79.3% vs. 78.1%), vessel analysis (77.8% vs. 77.6%), and surface pattern analysis (78.1% vs. 76.9%). However, the accuracy of color analysis in the interactive training group was better (74.4% vs. 80.0%).

Subgroup analysis is also important for predicting the pathology of colorectal polyps. For the after-training phase, the male trainees (79.7% vs. 71.0%, P = 0.014), the trainees with experience less than 500 cases (80.45% vs. 70.0%, P = 0.008), and the trainees with magnifying endoscopic experience less than 100 cases (80.71% vs. 72.08%, P = 0.020) in the interactive training group showed better accuracy of color analysis. It is possible that the interactice diccussion had more positive effect to decrease the confusion of color analysis than vessel pattern and surface pattern. No other statistical difference was discovered during the subgroup analysis between the two training groups.

The main limitation of this study is that the tests were based on still images that were collected from endoscopic databases of the five educational hospitals. The tests were not based on live demos or videos, which may offer more information about the lesions. Because of the COVID-19 pandemic, live demos and videos were difficult to collect. If possible, in a future study, we will investigate the outcomes of video or live demo-based tests.

In summary, in the after-training phase, self-driven training showed comparable accuracy of NICE classification, vessel pattern, and surface pattern but lower accuracy of color analysis. The male trainees, the trainees with experience less than 500 cases, and the trainees with magnifying endoscopic experience less than 100 cases in the interactive training group showed better accuracy of color analysis. The diagnostic performance of self-driven training in the prediction of colorectal polyp histology by trainees using the NICE classification is practicable and acceptable. Self-driven training is worth spreading during the COVID-19 pandemic.

## Data Availability

Data accessibility Supporting data can be accessed by email to qwg1991@126.com.
